# Knowledge, attitude and utilisation of evidence-based therapeutic exercises in knee osteoarthritis management in Nigeria

**DOI:** 10.4314/gmj.v58i1.13

**Published:** 2024-03

**Authors:** Adesola C Odole, Anita Okafor, Olufemi O Oyewole, Ezinne Ekediegwu

**Affiliations:** 1 Physiotherapy Department, University of Ibadan, Ibadan, Nigeria; 2 Physiotherapy Department, Olabisi Onabanjo University Teaching Hospital, Sagamu, Nigeria; 3 Astella Physiotherapy Clinics, Enugu and Nnamdi Azikiwe University, Anambra, Nigeria

**Keywords:** Therapeutic exercise, evidence-based practice, knowledge, attitude, utilisation, Nigeria Physiotherapists

## Abstract

**Background:**

In Nigeria, there is a disparity among physiotherapists regarding therapeutic exercise as a core treatment for patients with knee osteoarthritis (OA). The attitudes and beliefs of physiotherapists could influence this.

**Objective:**

To investigate Nigerian physiotherapists' knowledge, attitude, and utilisation of evidence-based therapeutic exercises

**Design:**

A mixed-method of cross-sectional survey and focus group discussion.

**Setting:**

Secondary and tertiary health institutions in Nigeria

**Participants:**

Physiotherapists consecutively sampled from the selected institutions.

**Main outcome measures:**

Participants' knowledge, attitude and utilisation of evidence-based therapeutic exercises for the management of knee OA

**Results:**

This study revealed that 81% of physiotherapists in Nigeria had a fair knowledge of evidence-based practice and the efficacy of therapeutic exercises in managing knee OA. Despite this fair knowledge, 95.3% had a poor attitude. The important emerging categories/themes are treatment preference, clinical experience, and strength of evidence.

**Conclusion:**

Physiotherapists in Nigeria have a fair knowledge of evidence-based therapeutic exercises in managing patients with knee OA, although there is a poor attitude and disparity between the use and current recommendations.

**Funding:**

The research received no funding from a commercial or non-profit organisation

## Introduction

Osteoarthritis (OA) is one of the disabling conditions which may ultimately lead to joint failure and significant disability.^1^ It poses physical, psychological and socioeconomic burdens on the sufferers.^2^ It is associated with significant disability, such as a reduction in mobility, pain and activities of daily living, sequel to its psychological burden, distress, devalued self-worth, and loneliness.^2^ The global prevalence of OA is known to increase with age 3, with almost one-third of individuals aged 40-60 years having radiographic evidence of the disease.^4^,^5^ It is estimated that over 10% of the global population above 60 years is affected by OA.^6^ Its prevalence is higher in women (18%) than men (10%).

Clinical guidelines and systematic reviews consistently recommend exercise as a core treatment for patients with knee OA.^6^–^9^ Evidence-based recommendations on therapeutic exercises for the management of OA include aerobic exercises, strengthening exercises, and aquatic exercises.^10^

The best mode of management for knee OA is a combination of aerobic and strengthening exercises, which have been proven to relieve pain and improve function in patients with knee OA.^9^

Physiotherapists are the most appropriately trained healthcare professionals with the knowledge to deliver exercise advice.^11^ Despite the adequate knowledge of clinical guidelines about therapeutic exercise in OA management, clinicians' adherence to these guidelines is suboptimal.^12^,^13^ Their attitudes and beliefs often influence this behaviour of healthcare professionals. In addition, patients' preferences and beliefs, professional and organisational factors, clinical applicability, and lack of time and language have been observed to determine therapeutic exercise use.^12^,^14^,^15^ Their knowledge, attitude and beliefs influenced the utilisation of evidence-based therapeutic exercises by physiotherapists in the United Kingdom about therapeutic exercises.^16^,^17^

It is unclear whether the clinical practice of physiotherapists in Nigeria entails the utilisation of evidence-based therapeutic exercises in their management of patients with knee OA. Moreover, the knowledge and attitude of physiotherapists in Nigeria on the utilisation of specific therapeutic exercises in line with evidence-based practice have not been investigated. This study was, therefore, designed to describe the knowledge, attitude and utilisation of evidence-based therapeutic exercises among physiotherapists in Nigeria.

## Methods

### Study population and design

A mixed-method study (cross-sectional survey and a focus group discussion (FGD)) was carried out between 2015 and 2016. Physiotherapists practising within Nigeria with at least two years of experience and who reported treating at least one patient with knee OA in the past 6 months were drawn consecutively. Before the data collection, the sample size was determined to assume that 9% utilised therapeutic exercise only in previous studies with a precision/absolute error of 5% and a type 1 error of 5%.^16^,^18^ A minimum of 126 physiotherapists was calculated to power the study. The purposive sampling technique was used to recruit 10 physiotherapists for the focus FGD.^19^ One session of FGD was conducted until data saturation was achieved.

### Instruments

#### Questionnaire on physiotherapists' knowledge and utilisation of therapeutic exercise in knee osteoarthritis

It is a 37-item, 3-part, researcher-developed questionnaire. The questionnaire assessed physiotherapists' knowledge and use of therapeutic exercises in the management of knee OA. Items on the questionnaire were devised based on a literature review on evidence-based therapeutic exercises and the recommendations of therapeutic exercises in the management of knee OA.^6^,^7^,^9^,^20^,^21^ Section A: contains eight items on socio-demographic information of participants.

Section B contains 14 items on physiotherapists' use and knowledge of evidence-based therapeutic exercises in managing knee OA. This section has two parts. Part I contains eight Items on the use of evidence-based therapeutic exercises and six items on knowledge of the strength of evidence of therapeutic exercises.

Section C: contains 15 items on physiotherapists' general knowledge of evidence-based therapeutic exercises in managing knee OA. The response options are Yes, No, and I do not know.

#### Scoring

Knowledge was scored in percentages. A correct (yes) response had a score of 1, while No, I do not know, was scored zero. The maximum obtainable score was 21, and the minimum obtainable score was 0. Knowledge score was transformed in percentages as [(Participants score/21) X 100]. Level of Knowledge was categorised as 0 to ≤ 49% (Poor knowledge), 50 to ≤ 79% (Fair knowledge), and 80 to 100% (Good knowledge). The questionnaire was validated by 6 Physiotherapy lecturers (knowledgeable in questionnaire development and validation).

#### Questionnaire on physiotherapists' attitude regarding exercise and knee osteoarthritis

It was developed by Holden et al 16 to assess physiotherapists' attitudes regarding exercise and knee OA. It is a 23-item, 2-part questionnaire rated on a 6-point Likert scale ranging from 1 to 6, where 1 is totally disagrees, 2 largely disagrees, 3 minimally disagrees, 4 is neutral, 5 is largely agreed, and 6 totally agrees. It contains 12 attitudinal statements on the perceived benefits of therapeutic exercises and 11 attitudinal statements on exercise delivery and adherence.

Scoring: Attitudinal items stated in a negative form with correct response of disagreement was scored between (Totally disagree-6, largely disagree-5, minimally disagree-4, Neutral-3, largely agree-2 and totally agree-1). Items stated in positive form with correct responses were scored as (Totally agree-6, largely agree-5, Neutral-4, minimally disagree-3, largely disagree-2, and totally disagree-1). The maximum and minimum obtainable score is 138 and 23, respectively. Attitude score was transformed to percentages: [(Score/138) X 100]. The score was categorised as 60-100 (Positive) and 0-59 (Negative).

#### Focus Guide

The focus guide (Appendix II) was developed through a literature review of studies on physiotherapists' use and knowledge of evidence-based therapeutic exercises in the management of patients with knee OA. The focus guide had five questions that served as a guide.

#### Procedure

Ethical approval was granted by the University of Ibadan/University College Hospital Ethics Research Committee (approval number: UI/EC/15/0469). Before the questionnaire was administered, a filter question was asked to screen physiotherapists who reported treating at least one patient with knee OA in the last six months. Questionnaires were distributed by hand or through a contact person and collected via the same means.

The FGD session was analysed thematically. It was guided by a moderator who was knowledgeable about focus group discussion. Participants were purposively sampled according to the pre-determined inclusion criteria. Anonymity was ensured throughout the discussion as participants were identified with numbers. The session lasted for 90 minutes. The FGD was carried out at the outpatient physiotherapy clinic of a tertiary health institution.

#### Data analysis

Data analysis was carried out using the Statistical Package for Social Scientists, version 23. Content and thematic analysis based on grounded theory were carried out on the data from the focus group discussion. Descriptive statistics of mean, standard deviation and percentages were used to summarise the data, while inferential statistics of Chi-square were used to determine the association between outcomes variables and socio-demographics. Alpha level (α) was set at 0.05.

## Results

**Figure 1:**
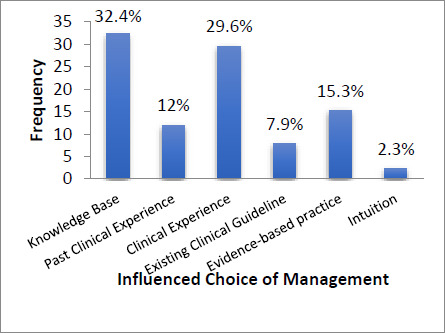
Distribution of Bases for Participants' Choice of Exercise in Knee in OA Management

Four hundred questionnaires were distributed; 216 copies were properly completed, with a response rate of 54%. Characteristics of participants are presented in Table 1. Physiotherapists utilised one form of therapeutic exercise in their management of patients with knee OA in combination with heat and ice; however, 48.6% utilised evidence-based therapeutic exercises. Figure 1 shows the basis for participants' choice of exercise in knee OA management. 62% based their choice of exercise on either knowledge base or clinical experience, while a few 15.3% based their choice on information from evidence-based practice. 67.1% utilised their treatment approaches very often, while 7% used these approaches sometimes. A combination of aerobic, range of motion, isometric and squatting exercises was commonly used (27.3%).

**Table 1:** Socio-demographics of participants

Characteristics	Cross-sectional n(%)	Focus group n(%)
**Marital Status**		
**Married**	125(57.9)	8(80)
**Single**	91(42.1)	2(20)
**Total**	216(100.0)	10(100.0)
**Clinical experience (years)**		
**0-4**	86(39.8)	
**5-9**	107(49.5)	
**10-14**	10(4.6)	
**15-19**	13(6.1)	
**Educational Status**		
**Bachelor degree**	162(75)	1(10)
**Master of Science**	46(21.3)	8(80)
**Doctor of Philosophy**	8(3.7)	1(10)
**Rank**		
**Physiotherapist**	134(60.0)	3(30)
**Physiotherapist1**	6(2.8)	0(0)
**Senior Physiotherapist**	50(23.1)	4(40)
**Principal Physiotherapist**	18(8.3)	1(10)
**Chief Physiotherapist**	3(1.4)	2(20)
**Assistant Director of Physiotherapy**	5(2.3)	0(0)
**Gender**		
**Female**	83(38.4)	5 (50)
**Male**	133(61.6)	5(50)
**Geopolitical distribution**		
**South-east**	38(17.6)	
**South-south**	19(8.8)	
**South-west**	89(41.2)	10(100)
**North-west**	26(12)	
**North-east**	15(6.9)	
**North-central**	29(13.4)	

**Figure 2:**
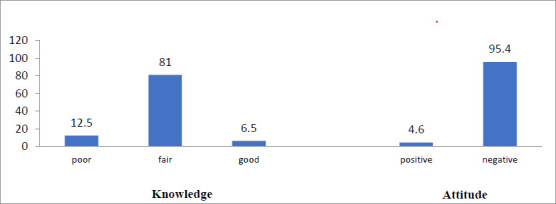
Participants' overall knowledge and attitude on evidence-based therapeutic exercise in knee osteoarthritis

Figure 2 shows the participants' knowledge and attitude to evidence-based therapeutic exercises. A large proportion of the participants (81%) had a fair knowledge of evidence-based therapeutic exercises in the management of knee OA. Few (12.5%) had poor knowledge, while only 13 (6.5%) had good knowledge. 95.3% had a negative attitude, while 4.7% had a positive attitude to evidence-based therapeutic exercises in the management of knee OA.

Participants' responses to attitude statements on perceived benefits of exercise revealed that 154 (71.3%) agreed “general exercises improve knee problems”, 160 (74.1%) “knee problems are improved by strengthening exercise”, and that “Physiotherapists should prescribe local strengthening exercises for every patient with knee OA” (63.4%). No consensus was reached regarding these statements about “Physiotherapist should prescribe general exercises for patients with the knee” (43.1%), “Exercise works as well for everyone regardless of the pain they have” (36.2%), exercise is effective for patients if X-ray shows severe knee OA (33.8%), “General exercise is safe for everybody to do (43.1%). Table 2 further represents a summary of participants' responses.

**Table 2:** Participants' response to attitude statements on perceived benefits of exercise in knee osteoarthritis and exercise delivery and adherence

Questions	Totally disagree n(%)	Totally agree n(%)
**Knee problems are improved by general exercises**	62(28.7)	154(71.3)
**Knee problems are improved by local strengthening**	56(25.9)	160(74.1)
**Physiotherapists should prescribe general exercise for patients with knee OA**	123(56.7)	93(43.1)
**Physiotherapists should prescribe local strengthening exercises for patients with knee OA**	79(36.6)	137(63.4)
**Exercise works as well for everyone regardless of the pain they have**	138(63.8)	78(36.2)
**Exercise is effective for patients if an X-ray shows severe knee OA**	143(66.2)	73(33.8)
**Exercise is effective for patients if an X-ray shows moderate knee OA**	96(44.5)	120(55.5)
**Exercise is effective for patients if an X-ray shows mild knee OA**	67(31)	149(69)
**Increasing overall activity level stops the knee problem from getting worse**	114(52.7)	102(47.3)
**Increasing the strength of muscle around the knee stops the knee problem from getting worse**	82(44.5)	134(62)
**General exercise is safe for everybody to do**	163(56.9)	53(43.1)
**Local strengthening exercises for the knee are safe for everybody to do**	107(49.5)	109(50.5)
**It is the patient's own responsibility to continue doing their exercise program**	94(43)	122(56.5)
**It is the physiotherapist responsibility to make sure that patient will continue doing exercise program**	94(43)	122(56.5)
**How well a patient complies with their exercise program determines how effective it will be**	51(23.6)	165(76.4)
**The physiotherapist is the best person to decide whether patient should do their exercise at home or in group setting**	86(39.8)	130(60.2)
The patient is the best person to decide whether a patient should do their exercise at home or in group setting	166(76.9)	50(23.1)
**Exercise for knee OA is the most effective if done as a home program**	89(41.2)	127(58.8)
**Exercise for knee OA is most effective if done in a group setting**	169(78.2)	47(21.8)
**It is important that people with knee OA increase their overall activity levels**	93(43)	123(57)
**Physiotherapist should educate chronic patients with knee OA about how to change their life style for the better**	38(17.6)	178(82.4)
**A standard set of exercises is sufficient for every patient knee OA**	77(35.6)	139(64.4)
**Exercise for knee OA is most beneficial when tailored to meet individual patient needs**	22(10.2)	194(89.8)

Participants' responses to attitude statements relating to delivery and adherence to exercise also showed that there was consensus among 89.8% and 82.4% of the participants on the attitude statements that “exercise for knee OA is most beneficial when it is tailored to meet individual patient needs” and “physiotherapists should educate chronic patients with knee OA about how to change their lifestyle for the better” respectively. However, no consensus was reached regarding the statement, “the patient is the best person to decide whether they should do their exercises at home or in a group setting” (23.1%), “exercise is most effective if done in a group setting” (21.8%). Although 56.5% and 58.8% agreed, “it is the physiotherapist's responsibility to make sure patients will continue doing their exercise program” and “exercise for knee OA is most effective if done as a home program”. Participants' responses are further presented in Table 2.

The association between knowledge and participants' demographic characteristics shows no significant association. There was no significant association between participants' knowledge and gender (p=0.242), age group (p=0.294), and years of clinical experience (p=0.145). There was no significant association between participants' attitude and their educational status (p=0.410), years of clinical experience (p=0.940), gender (0.182), and age (0.768). There was a significant association (p=0.007) between respondents' knowledge and attitude.

### Focus group results

Four categories emerged, each of which contained several themes.

#### Participants' knowledge of evidence-based practice in knee osteoarthritis

The question “How do you manage knee OA?” was used to explore participants' knowledge of evidence-based therapeutic exercises for knee OA management. Probes were used to explore this category further. Four themes were generated: knowledge based on clinical judgment and clinical experience, knowledge based on research/published works, lack of information about evidence-based therapeutic exercise, and lack of updates.

***Knowledge based on clinical judgment and clinical experience: Participants' response to their knowledge of evidence-based therapeutic exercises*** in the management of knee OA revealed that their knowledge was based on their clinical judgement and experience. They believe evidence is obtained from their practice over time, which, in their opinion, has resulted in positive treatment outcomes.

*“My choice of treatment in OA is majorly dictated from my experience, most patients with OA have inflammation of the synovium, most of them have inflammation around the joint, this means inflammation around the capsule of the joint and usually this is what guides my choice of cryotherapy as a modality to bring down the pain at the initial stage” (participant 9)*.

***Knowledge based on Research/Published Works:*** Some of the participants reported to getting their source about evidence-based practice from published works. This finding indicated that their knowledge about the evidence on knee OA management was premised on research findings of interventions that resulted in improved treatment outcomes.

*“my sources of knowledge are from published research, individual research, from my knowledge, one single research is not evident enough to either throw out or take in reports from studies, one still needs reviews from other studies” (participant 1)*.

***Lack of information about evidence-based therapeutic exercise:*** Participants also reported that lack of information about evidence-based therapeutic exercise limits their knowledge of this. They claimed that patients are just treated based on symptoms and not on evidence-based information since such information is not readily available.

*“Our practice of these days is very low, people do not update themselves about what is going on, and they base it on previous knowledge. A lot of people do not know about evidence-based practice, we just follow the norm, we have a traditional way” (participant 3)*.

#### Lack of Updates and treatment Paradigm

The finding that there are no updates on evidence-based therapeutic exercise in knee OA management and an existing treatment paradigm has limited participants' knowledge about evidence-based therapeutic exercise. It is assumed that physiotherapists get into the flow of the existing treatment pattern and do not bother to get updated on current trends in management.

*“it is because a lot of people do not update themselves about what is going on or what should be done, and they base it on previous knowledge, because they don't seek to know it and it is an area we must look into. A lot of people do not know what evidence-based practice means, we don't bother to read about it since there is norm in practice, that is what majority of us do, we have a traditional way” (Participant 3)*.

#### Physiotherapists' Attitude to Evidence-based Therapeutic Exercise

One theme was generated from this category: Treatment Preferences and existing Clinical practice behaviour. The majority of the participants reported that in the absence of cryotherapy and thermotherapy, they would not use exercises alone in managing knee OA. They opined that most patients with knee OA often present with pain, and as such, their plan of treatment must include the use of either cold or heat therapy. They are inclined to manage patients with OA based on what they know works for the patients.

*“not having to use either cryotherapy or thermotherapy in managing knee OA sounds somehow, with the level of pain patients presents with, for aerobics around here they need to shed their weight” (participant 3)*.

*“If we just decide that for all our patients with knee OA we would use only aerobics I think it will be somehow, considering the fact that such patients present with pain” (participant 4)*.

#### Physiotherapists' Utilisation of Therapeutic Modalities

Most Physiotherapists were concerned about the symptoms and pathology of knee OA. Three themes were generated from the category on utilisation of therapeutic modalities: Clinical experience, symptoms of knee OA, and availability of treatment modalities. Only one participant based his utilisation of therapeutic modalities on evidence and strength of evidence.

***Clinical experience:*** It was also highlighted that clinical experience also plays a role in managing patients with knee OA. Physiotherapists believe that therapeutic exercises are based on their clinical experience, especially when the interventions result in the improvement of symptoms. Participant 7 had this to say *“My choice of treatment of OA is majorly dictated from my experience, most patient with OA have inflammation of the synovium, most of them have inflammation around the joint, this means inflammation around the capsule of the joint usually and this is what guides my choice of cryotherapy as a modality to bring down the pain at the initial stage, and of course because of the pain which has been there for some time the patient has developed loss of range of motion and has developed muscle weakness so it is just natural you strengthen the quadriceps, weight bearing exercises again quite a number of them have weight bearing asymmetry…so usually those are the things that guides my own choice of intervention”*.

***Symptoms of knee OA:*** The emphasis on how knee OA is being managed by most of the participants was mmainly to reduce pain and swelling. Participant 1 had this to say *“Well they usually present with pain which is the most disturbing symptoms that patients present with, so usually we want to get the pain down by using a thermo therapeutic modality most likely hydropack or something, because that is what is available to us here, then after that soft tissue massage, strengthening exercises, quads and hamstrings then maybe some other circuit exercises may follow”*.

***Availability/non-availability of treatment modalities:*** Participant 1 had this to say *“most patient present with pain, first we try to bring the pain down with a modality like Hydrocollator pack which is what is available here, then we do soft tissue massage, strengthen muscles (Quadriceps and Hamstrings) and maybe some other circuit exercises”*.

#### Participants' Knowledge of Evidence-based Therapeutic Exercise in the Management of Knee Osteoarthritis

One theme was generated from this category: strength of evidence. Only one participant had adequate knowledge on the strength of evidence-based therapeutic exercises in the management of knee OA. Participant 7 stated “in *my practice and from my experience from research I guide what I do…a lot of things we actually do not have evidence to support them or very weak evidence. I have found both strengthening and aerobics are level 1, that is the highest level and that is why all our patients should have exercises…TENS has level 2-3 evidence*.

## Discussion

We investigated Nigerian physiotherapists' knowledge, attitude, and utilisation of evidence-based therapeutic exercises. Physiotherapists in Nigeria had fair knowledge and a negative attitude toward evidence-based therapeutic exercises in managing patients with knee OA. Although there is a disparity between the use and current recommendation, they utilised evidence-based therapeutic exercises in conjunction with other modalities. To a large extent, these observations may be influenced by the patient's perspectives and the practice environment.

### Utilisation of evidence-based therapeutic exercises in the management of knee osteoarthritis

All the participants reported that they utilised one form of therapeutic exercise or the other in this study. This finding was in agreement with those of Holden et al 16 in a study in the UK among physiotherapists. The authors reported that almost all (99%) of the physiotherapists utilised therapeutic exercises. All the participants in this present study agreed to utilise therapeutic exercises in managing patients with knee OA. They utilised aerobics, isometrics, range of motion, and squatting exercises, mostly in conjunction with either heat or ice. These findings are in agreement with the findings of Holden et al.^17^, who reported that participants also utilised therapeutic exercises with other approaches like acupuncture, heat, and massage in the management of knee OA.

This study revealed that none of the participants used evidence-based therapeutic exercises exclusively in managing patients with knee OA. The majority used a combination of different exercises. The unexclusive use of therapeutic exercises may be attributed to patients' perspectives or preferences.

Various reasons have been suggested for choosing treatments among patients with OA. Treatment characteristics, fear of pain, risks, joint damage, patients' confidence and mindset, belief about capability, personal investment, personal circumstances, and support and advice are key themes guiding treatment choices for knee and hip OA.^22^,^23^

Existing studies have proven that therapeutic exercises of aerobic, strengthening and aquatic exercises effectively manage patients with knee OA with strong and moderate evidence.^10^ However, the results from this study showed that physiotherapists prefer to prescribe a combination of treatment approaches (aerobic exercises, isometric, range of motion, squatting exercises, heat, ice and aquatic exercises), which is not in agreement with evidence-based practice.^10^ In a similar vein, Holden et al 17 also reported that physiotherapists preferred to prescribe local (strengthening) exercises to general (aerobics) exercises, though not in line with current recommendations for managing patients with knee OA. While we do not try to deprive physiotherapists of their knowledge, they should be encouraged to utilise evidence-based guidelines in knee OA management. In the absence of a national protocol, each centre can adopt established guidelines as their protocol in the management of knee OA for better outcomes.

### Physiotherapists' knowledge and attitude of evidence-based therapeutic exercises in knee OA

The findings from the qualitative and quantitative aspects of this study revealed that physiotherapists in Nigeria have a fair knowledge of evidence-based practice and that therapeutic exercise is effective in managing knee OA. Still, the majority, despite their knowledge of evidence-based practice, could not indicate the strength of evidence each of their treatment approaches had. This may indicate why only a small proportion (15.3%) reported that their choice of management was influenced by information from evidence-based practice, while 2.3% based their choice on intuition. In comparison, 32.4% and 29.6% reported their knowledge and clinical experience influenced their choice of management approaches. The majority of the participants in this study did not have post-graduate training. A large proportion (75%) in this study had no post-graduate training, which appears to have influenced their choice of evidence-based therapeutic exercises in the management of knee OA. A study 24 reported that physiotherapists with higher levels of training are more likely to search databases and understand a range of evidence-based practices; they also reported that 69.4% frequently read about research, but only 25.8% critically appraised the research reports. This implied that physiotherapists may have knowledge about evidence-based practice but may not apply this knowledge in their practice.

Data from the focus group discussion showed that the major aims of an exercise program for knee OA from the physiotherapists' perspectives in this study were to relieve pain, increase range of motion and strengthen muscles around the knee. This finding disagreed with that of Holden et al 17, who reported that participants in their study aimed to increase muscle strength around the knee, stabilising the joint rather than providing pain relief. This may explain why, within this study, the use of exercises in conjunction with either heat or ice was more commonly prescribed than local exercises (strengthening) or a general exercise (aerobics exercises), which is the mainstay in managing knee OA in line with current evidence-based practice. As shown from the findings in this study, exercise was related to increasing the severity of joint damage or pain level, with physiotherapists believing cryotherapy, heat therapy, and manual therapy as adjuncts to exercise are more effective than exercise alone. The themes from the focus group discussion offered some explanation for these findings. Discussants viewed knee OA from a biomedical perspective, attributing signs and symptoms to local knee joint pathology or wear and tear. OA was seen as a chronic degenerative condition that would progressively worsen over time; the best way of management was to reduce pain by using modalities like cryotherapy, infrared or hydro pack, even though studies have reported the efficacy of aerobic and strengthening exercises in the reduction of pain and improving function.

Despite physiotherapists' knowledge of evidence-based practice or therapeutic exercise, it seems they do not practice it, as 94.4% of them reported a negative attitude to the prescription of therapeutic exercises. Although research on evidence-based practice among physiotherapists has revealed that some barriers limit the utilisation of evidence-based practice.^12^,^14^,^15^

This presumably could be why the participants in this study had a negative attitude towards the use of evidence-based therapeutic exercises in the management of knee OA. There was a significant association between the knowledge and attitude of the participants in this study. The finding tends to be misleading considering the fact that the majority of the physiotherapists reported a fair knowledge and negative attitude to evidence-based therapeutic exercises in the management of knee OA. A plausible reason could be that most physiotherapists already have a paradigm in their usual practice of managing patients with knee OA. This may have been attributed to the negative attitude they reported towards the use of evidence-based therapeutic exercises in the management of knee OA. Anecdotally, in Nigeria, it appears there are no treatment guidelines or standard operating practices in knee OA management in most institutions. Another reason could be the finding that knee OA was viewed as a painful degenerative disease that exercises alone could exacerbate the symptoms of pain. Hence, they tend to avoid exercise in their management because they feel it will worsen the pain and degenerative process.^25^ Again, physiotherapists could be encouraged to prescribe therapeutic exercises positively by formulating standard operating practices in knee OA management that include therapeutic exercises in Nigerian health institutions.

### Perceived benefits of exercise for knee OA

The majority of the participants in this study agreed that “knee problems are improved by local (strengthening exercises)” and general exercises (aerobic exercises) for every patient with knee OA. This is in agreement with the previous study.^17^ Participants in this study disagreed about the safety of local or general exercise for every patient with knee OA. This may be a result of the fact that physiotherapists feel exercise may increase pain, though the majority agreed increasing muscle strength around the knee stops the knee problem from getting worse; this may be a result of reports from studies that have identified the efficacy of strengthening exercises in knee OA.^6^,^26^,^27^ The physiological effects of therapeutic exercises have been shown to confer benefits on pain and other symptoms of OA.

### Exercise delivery and adherence

There was consensus among survey participants that “exercise for knee OA is most beneficial when it is tailored to meet individual patient needs” and “physiotherapists should educate patients with chronic knee OA about how to change their lifestyle for the better” This is in agreement to the recommendations in the NICE guidelines as reported by Skou and Roos 8 who stated that physiotherapists are the most appropriately trained healthcare professionals with the knowledge to deliver exercise advice. A consensus was reached regarding the statement, “People with knee OA must increase their overall activity levels”. This may be due to the fact that physiotherapists believe overweight patients are likely to experience knee pain due to OA, so advising patients on their overall activity will be beneficial in their management.

#### Strength and limitation

The mixed-method design used to collect data seems to provide in-depth findings about the subject. However, the information provided by respondents could have been limited by a bias in responding, referred to as faking good, as some of the participants could have intentionally attempted to create a false positive impression about their knowledge, perception and utilisation of evidence-based therapeutic exercises in the management of knee OA. The convenient sample may limit the power of generalisation.

## Conclusion

Physiotherapists in Nigeria had fair knowledge and a negative attitude toward evidence-based therapeutic exercises in managing patients with knee OA. They utilised evidence-based therapeutic exercises in conjunction with other modalities, and no physiotherapist has used exercises (aerobics and strengthening) exclusively to manage patients with knee OA. The findings of this study suggest that good knowledge of evidence-based practice does not translate to a positive attitude in utilising evidence-based therapeutic exercises in the management of patients with knee OA.
